# Hôtel Dieu

**Published:** 1830-01-01

**Authors:** 


### CLINICAL REVIEW.

### L.

### HOTEL DIEU.

### 1. Rhinoplastic Operation for Destruction of the Lower Lip.*

The rhinoplastic, or, as it is generally termed in this country, the Taliacotian operation is applicable, no doubt, to many more cases of destruction of the superficial parts, than of that most prominent feature of the "human face divine" to which it is adjudged par excellence. Sir Astley Cooper, we all know, healed a fistula in perineo by a flap from the scrotum, and Mr. Earle followed in Sir Astley's wake on a similar occasion and with similar success. M. Dupuytren has recently applied the same principle to the cure of the deformity and destruction produced by that horrible malady, the cancrum oris.

*Case.* A male child, 11 years of age, was attacked, about eighteen months ago, with cancrum oris, which destroyed the half of the lower lip of the right side, from the median line to the inferior border of the lower jaw, and a portion of the cheek to near the angle of the maxilla. On admission into the Hôtel Dieu, the loss of substance of the cheek extended very little above the level of the commissure of the lips, was bounded behind by the inferior border of the masseter muscle, and reached below to the lower edge of the maxilla, which was there in a state of caries. The left half of the jaw, which had lost the point d'appui that the symphysis naturally affords, was dragged inwards by the action of the muscles, so that the row of teeth on this side was applied to the arch of the palate. This portion of the jaw, however, continued moveable, and could be readily returned to its proper situation. The aspect of the child was disgusting in the extreme, the tongue in part hanging out from the centre of the chasm, in part adhering by its right border, which greatly embarrassed its movements; the saliva constantly running out; mastication very imperfect, and deglutition painful. The health, notwithstanding all this, was good, and the lad lived entirely on soups and soft food. He was anxious to be rid of his deformity, and promised to suffer any thing for that purpose. M. Dupuytren commenced by destroying the unnatural adhesions of the right side of the tongue with a bistoury; but the essential part of the operation remained behind, and required mature deliberation

* Journ. Hebdomad. No. 56.

before deciding finally on the mode to be pursued. Two or three plans were suggested to the able Baron, but we shall only notice that which was actually adopted. The object was to remove a flap of sufficient size from the neck, apply it to the gap, and retain it in its new situation by the twisted suture. The operation was performed on the 31st of August.

Having traced with ink the dimensions and form of the flap, which he determined to procure from the lateral, superior, and steno-cleido-mastoidean portion of the neck, M. Dupuytren dissected it off, taking care not to wound the jugular vein. The edges of the excavation having then been pared, the flap was twisted on the narrow band that still connected it with the parts in the neck, the edges placed in apposition with the newly pared ones of the opening in the cheek and lip, and both retained in due connexion by the employment of the twisted suture in five places. The sides of the integument in the neck were also reunited by three sutures, two little arteries which poured out their blood were secured, and no other dressing was employed. The operation was one of much delicacy and occupied a considerable time, but the little patient bore it with great courage.

All went on favourably till the 2d of September, on which day the flap had not lost its vitality, but was even suppurating at one or two points of its circumference. In the night, however, of the 2d or 3d of September restlessness and delirium supervened, and the patient tore away one of the needles which united the edge of the lower lip with the anterior portion of the flap. A separation between the two for an inch in length and half an inch in breadth was the consequence, and adhesive straps were applied by M. Dupuytren to bring the parts together. Next night, the fever and delirium continuing, a second needle, uniting the base of the lip with the inferior anterior portion of the flap, injured and tore the parts, which in the morning appeared to be slightly sphacelated. Nevertheless the flap did not die, but had by this time contracted solid adhesions above and behind. On the 4th M. Dupuytren removed all the needles and maintained the necessary degree of apposition by adhesive straps. On the 5th the unfavourable symptoms had disappeared, and the flap adhered extensively and firmly, though portions furnished a little suppuration. The laceration in front became a simple hare-lip fissure, which might be, and in point of fact very shortly was, treated by the usual operation for that deformity by M. Dupuytren. It failed, however, in consequence of smart hæmorrhage and consequent disturbance of the dressings occurring on the following morning, and the hare-lip of course continued. On the 12th of October the union of the flap and neighbouring parts was perfect at all other points, its vitality was also perfect, the wound in the neck was quite cicatrised, and as the surgeon thought the patient would be better able to undergo the hare-lip operation when his system had been invigorated by time and fresh air, he was dismissed the hospital, and ordered to return at a future opportunity.

Had it not been for the accidental violence inflicted on the lip, it is more than probable that the union of the flap with the neighbouring parts would have been complete. As it is, the diminution of the deformity in this poor child must be very considerable, and the results of the operation are calculated to encourage the surgeon in the application and extension of the rhinoplastic operation to many cases, in which its perfomance is at present never dreamt of.

### II. ENCYSTED HYDROCELE OF THE SPERMATIC CORD.

There are some in the profession who profess that they find no difficulty in the diagnosis of hernia, and look with an air somewhat bordering on contempt on those who are more honest or less fortunate than themselves. For our own parts, we must own that we have met with difficulties, and seen them occur to very able surgeons. It is well known, at least to those who know any thing about the matter, that encysted hydrocele of the cord has been a prolific source of error, and Pott and Cooper, the surgeons of some of our London Hospitals, and of the Glasgow Infirmary, with many others whom we might name in private practice have found to their cost that it was so. We have no intention of commencing a studied disquisition on the subject, or deploring, like Peter Pindar's pilgrim,

"With outstretch'd bum and bended knees,"

the backslidings of our brethren or our own. We are glad, however, that a case and a clinical lecture from M Dupuytren, will enable us to give that admirable surgeon's observations on this frequently obscure affection. We conceive that we are doing an acceptable service to the profession, by pointing out from time to time the dangerous parts and holes in the scientific ice, into which the heedless may chance to tumble.

*Case.** A male child, about 12 years of age, had the operation of injection performed at the Hôtel Dieu in 1828, for common hydrocele of the left side. He was perfectly cured, but some months afterwards a small, soft, indolent, and fluctuating tumour appeared in the groin, opposite the external ring. It was considered to be a hernia and a truss was employed, but the tumour continued to increase, and the patient again entered the Hôtel Dieu in 1829. Opposite the external ring was a roundish tumour slightly ovoid, the size of a large pigeon's egg, commencing within half an inch of the ring, and terminating near the epididymis. Although pretty tense it communicated a sense of fluctuation, and the colour of the skin was unaltered ; it was evidently transparent ; received no impression during coughing ; and, though capable of being returned into the inguinal canal, it appeared to be circumscribed and distinct. From this assemblage of characters M. Dupuytren recognized an encysted hydrocele of the cord, and performed the operation of incision on the 13th of October. The patient for this purpose was placed upon his back, and an incision very cautiously made through the integuments covering the tumour. The subjacent layers of fascia or cellular membrane were then successively and carefully divided till the cyst was opened into, which was indicated by a jet of yellow serum. The opening was enlarged with the bistoury and scissors, the finger introduced into the sac to ascertain that it possessed no outlet, the contained serum entirely evacuated, and its cavity stuffed with lint in order to induce suppuration and granulation. Every thing succeeded as favourably as could be wished, and on the 20th, the date of report, the wound was nearly cicatrized.

M. Dupuytren observes that very great caution is frequently requisite in forming the diagnosis between this affection and hernia. By making the patient lie upon his back we perceive that the tumour is roundish, circumscribed, isolated, and distinct from the intestinal canal and omentum, besides which it is transparent, and commonly is attended with fluctuation. In spite, however, of these characteristic symptoms the diagnosis is often extremely difficult, and M. Dupuytren has witnessed " hundreds of cases " which had been mistaken for hernia and treated with a truss. In one patient in whom this error was committed, the tumour being constantly pressed upwards had mounted along the inguinal canal, and acquired a very great volume in the iliac fossa. With regard to the treatment, M. Dupuytren believes that injection would be the best, were we always certain of the nature of the disease. If, however, by mistake, a hernial sac were injected and the stimulated fluid thrown into the cavity of the peritoneum, the consequences may be readily imagined. In a Parisian hospital where operations are seldom practised, injection was performed in two cases of hydrocele of the tunica vaginalis, in which the communication with the abdomen continued open. One patient escaped, but the other died of peritonitis. Whenever then the slightest doubt of the nature of the cases exists, incision must

* Journ. Hebdomad. No. 58.

be practised instead of injection, and even this must be done with caution lest the vessels of the spermatic cord should be wounded. In the present instance such caution was adopted by M. Dupuytren, partly for the reason already alluded to, and partly from the bare possibility of the existence of a hernia.

III. Vesico-Vaginal Fistula.

*Case.** A woman, ætatis 30, who was affected with vesico-vaginal fistula supervening on a long and difficult labour, applied at the Hôtel Dieu in the course of last Winter, and was successfully treated by the actual cautery. Scarcely, however, had the urine ceased to flow through the fistulous opening, when the patient left the hospital and returned to her usual occupations. For some time she went on well enough, but getting into a voiture one day in a hurry, she felt something suddenly give way, the cicatrix in fact burst, and the fistula with all its train of inconveniences was forthwith reproduced. She now returned to the Hôtel Dieu, with the urine flowing involuntarily through the vagina, but not constantly or always in the same degree; if making exertion, little was discharged, but when lying still, the flow was abundant, and on trying to make water, the greater quantity issued through the fistulous opening. On examining by the finger, the opening was felt at the neck of the bladder and on the right side of the vagina; on employing the speculum its direction was perceived to be transverse, and it was formed of two lips, the superior of which, when the patient assumed the erect posture, was depressed, and passing a little below the inferior completely closed the fistulous orifice. This sufficiently accounted for the cessation of the involuntary discharge of urine on the patient's standing up, and its re-appearance on her lying down, the superior lip of the fistula becoming in the latter position the posterior one, and no longer offering a barrier to the discharge. M. Dupuytren determined, under these circumstances, to try whether the plan that had succeeded before might not succeed again; and on the 13th of October the operation of cauterization was performed.

The patient was laid upon her face across the bed, a pillow placed under her belly, and her thighs and legs allowed to hang out of the bed. The fistula was then brought fairly into view by means of the speculum, and the edges gently touched by a cauterizing iron raised to a white heat, and made in the shape of a French-bean. The pain and inflammation that ensued were relieved by a warm-bath in the course of the day, and up to the date of the report no discharge of urine by the fistula had taken place.

Vesico-vaginal fistulæ, says M. Dupuytren through the reporter, are not very uncommon after tedious labours, and constitute one of the most troublesome and disgusting infirmities to which the female sex is liable. The treatment generally pursued has been that recommended by Dessault, viz. the retention of a catheter in the urethra and the introduction of a plug of some kind into the vagina, in order to keep the edges of the fistula as much as possible in apposition. This plan though sometimes successful, has more frequently failed, and is extremely tedious at the best. M. Dupuytren has employed the actual cautery in these cases for a considerable time and with great success. He prefers it on every account to the use of the nitrate of silver, and employs it in the following manner:—the patient is placed across the bed upon her belly, with a pillow or two beneath her to elevate the pelvis, and her lower extremities out of the bed and held by assistants. A speculum, in two pieces and hollowed like a flute, is introduced into the vagina, and the fistula exposed to view. With a cautery shaped like a French-bean, named by M. Dupuytren *cautère en haricot*, the edges of the fistula are lightly touched, so as merely to stimulate without destroying them. The swelling which succeeds this cauterization chokes up the fistula for the time, the urine ceases to escape through the aperture, and either cicatrization and obliteration are

* Journ. Hebd. No. 58.

effected or the aperture is much contracted in diameter. Two or three applications of the iron are commonly required, and in order to ensure the free discharge of the urine from the bladder during the process a catheter may be kept in the urethra. This, however, according to M. Dupuytren, is seldom required, and the operation has succeeded in a great number of instances at the Hôtel Dieu. The most favourable cases are those in which the aperture is longitudinal, the most unfavourable when it is transverse. In the latter, when accompanied with much loss of substance and a considerable communication between the bladder and vagina, cauterization will scarcely succeed, and it then becomes necessary to resort to other means.

We think the foregoing observations on vesico-vaginal fistulæ worthy the attention of our brethren. Before concluding this article, we may allude to a clinical lecture published by that active and intelligent surgeon, Mr. Earle, in a late number of our contemporary, the Medical Gazette.* The lecture in question was delivered on the case of a woman in St. Bartholomew's Hospital, affected with a very extensive vesico-vaginal fistula, supervening on a long and ill-managed labour. Mr. E. does not seem to be aware of the use of the actual cautery in such cases; indeed, he makes no mention of the employment of the nitrate of silver, potassa fusa, or other means of stimulating the callous edges of the fistulæ, that have at various times been adopted. The following remarks deserve notice:

"In calculating the probability of success from any curative operation, much must depend on the situation and extent of the injury. When that portion of the bladder has perished which is situated in the triangular space included between the termination of the ureters and the commencement of the urethra, I fear no rational grounds of hope can be entertained of success, as the urine will flow through the fistulous opening as rapidly as it is secreted, and will effectually interrupt any hea ing process. Again, when the opening is in this situation, there would be danger of cutting across the termination of the ureters in endeavouring to remove the callous edges of the opening. This danger, and the difficulty of treating such a case, will be better understood by referring you to this preparation, and this drawing, which was taken from it when in a recent state. The subject from whom this preparation was removed was a young Irish woman, who came into the hospital in an advanced stage of acute peritonitis, and died the following day. She had been delivered one month, and had been attended by a drunken midwife, who suffered her to linger many days in labour, administering from time to time her favourite cordial to the patient and herself. In this case you will perceive that nearly the whole portion of bladder between the ureters and urethra has sloughed away. The bristles which are introduced into the ureters are seen immediately at the edges of the opening. It would have been quite impossible in this case to have effected any thing by operation. In this case you will likewise see the hernia of the mucous lining of the bladder, which is turned upwards, and is united to the neck of the uterus, just as I have described to be the case in the patient at present in the hospital. In the drawing the comparative vascularity, and other characteristics of the lining of the bladder and vagina, are clearly marked. In this case, also, you will observe that the mouth of the uterus has been closed by the inflammation which was produced. The uterus was distended with menstrual fluid, and it is probable that the peritonitis was excited by this obstruction, as there were much greater traces of inflammation over the pelvic viscera than in other parts of the cavity of the peritoneum. This circumstance reminds me that I had omitted to mention that in the case now in the hospital the patient has not menstruated since her confinement; but I have ascertained that there is no obstruction in the mouth of the uterus, by introducing a full sized

* For Nov. 14th, 1829.

bougie into its cavity. When the opening in the bladder is situated nearer the fundus beyond, or rather higher than the ureters, there is a far better prospect of success, as in that case there will be a cavity to receive the urine, which may be allowed to flow off through a short catheter as rapidly as it is secreted. The probability of affording relief even in such a case, must, however, depend on the extent of the opening, and the degree of eversion or hernia of the bladder; and here I would observe that this eversion of the bladder appears to depend on the greater destruction of the coats of the vagina in proportion to those of the bladder. In the process of cicatrization, where there has been a large slough of the vagina, the bladder is drawn out to supply its place, and becomes firmly united and continuous with the surrounding vagina. It must be confessed, that under the most favourable circumstances, these cases present the greatest obstacles, and are certainly the most difficult that occur in surgery. I do not mention this to discourage you from making attempts to relieve patients suffering under this great calamity; on the contrary, I would strongly urge you not to abandon them, and not to be deterred by many failures. I have succeeded in perfectly restoring three such cases; in one of which I performed upwards of thirty operations before success crowned my efforts."

The continual flow of urine, narrow space for operation, great sensibility of the parts engaged, and their disposition to slip from before the instrument, the impression made on the pelvic viscera by an accidental cough or sneeze, &c. and lastly, the great indisposition of mucous surfaces to take on the adhesive inflammation, are serious difficulties for the surgeon to contend with. Nil desperandum must be his motto, and to aid him in accomplishing the desired and desirable ends, Mr. Earle lays down the following directions:—

"It remains for me yet to state what curative and what palliative means we possess in these cases. When the opening is not situated between the urethra and ureters, or in the neighbourhood of the latter tubes; when it is not of great magnitude, and when there is not much hernia of the bladder, you may attempt to remove the callous edges, and unite them by the assistance of sutures. You will be much facilitated in this operation by previously dilating the urethra sufficiently to admit the fore-finger of the left hand; by which you will be enabled to draw down the bladder and to afford a support and resistance in removing the edges. The instruments best adapted for this purpose, are very narrow double-edged scalpels, or lancets, with which you may pierce through the membranes and cut your way outwards. These should only cut a short distance from the extremity. It will be better to commence at the extreme edges of the opening, and not to attempt to effect too much at any one operation. By several operations you will gradually diminish the aperture, but by attempting too much you will be foiled altogether. To enable you to convey a suture through the edges, to hold them in contact, it will be necessary to employ *port-aguilles*, with grooves which will hold a short triangular glover's needle at different angles, and with slides adapted for holding or letting loose the needle. The following is the mode in which I have employed this:—An armed needle should be fixed at the angle most convenient for piercing the denuded edges of the wound, which should be directed by the finger, and carried through the two edges. The point should be received by the other *port-aiguilles*, and the slide pushed up to fix it. The slide of the first should then be drawn down, which will leave the needle in the grasp of the second, by which it may be drawn through with its thread attached. To effect this in so narrow a space as the vagina, is often most difficult, and requires much patience and dexterity. The ligature should be drawn tight, and the ends cut off. I have also employed short hare-lip pins, and the twisted suture; but these are still more difficult to pass, and cause much more irritation. In those cases which, from the situation of the opening, or its magnitude, no curative means can be attempted, a well-adapted truss, with an elastic gum pad, will often enable the patient to retain a considerable quantity of water, and to enjoy comparative comfort."

Here we must stop for the present, and we again recommend our readers to peruse the observations of the French and the British surgeon on this very interesting, because very practical, subject.

